# Editorial: The relevance of core outcome sets to clinical guideline development

**DOI:** 10.1002/gin2.12026

**Published:** 2024-08-20

**Authors:** Sarah Rhodes, Paula Williamson, Ivan D. Florez

**Affiliations:** ^1^ Centre for Biostatistics, Division of Population Health, Health Services Research and Primary Care University of Manchester Manchester UK; ^2^ Department of Health Data Science, MRC‐NIHR Trials Methodology Research Partnership University of Liverpool Liverpool UK; ^3^ School of Rehabilitation Science, Faculty of Health Sciences McMaster University Hamilton Canada; ^4^ Department of Paediatrics Universidad de Antioquia Medellin Colombia

1

A core outcome set (COS) is ‘an agreed standard set of outcomes that should be measured and reported, as a minimum in trials for a specific health condition’,^1^ developed via consensus with stakeholders. Although the focus on COS originated on randomised trials, their use in systematic reviews, routine care, audit and, importantly, in clinical guidelines, is being increasingly recognised. The core outcome measures in effectiveness trials (COMET) initiative is an organisation for anyone interested in the development and application of COS and they host a database of studies relating to COS (https://comet-initiative.org/Resources/Database).

There are at least three main reasons why we would advocate the use of a relevant COS when developing a clinical guideline. First, there is the desire to have a research ecosystem where this minimum set of outcomes considered and reported in trials, combined in systematic reviews and used for clinical decision‐making and monitoring patient progress are the same. Consistency will reduce bias and increase efficiency, and COS are a means to facilitating this. Second, it reduces duplication of effort. COS developers go through a rigorous, transparent process and involve all relevant parties including patients and members of the public in determining the outcomes of critical importance;^1^, ^2^ guideline authors commonly repeat an almost identical procedure. Third, using COS in guidelines would encourage researchers to consider using them when designing trials.

The International Guideline Development Credentialing and Certification Programme (https://inguide.org/) mentions COS and some guideline organisations, including National Institute for Clinical Excellence (NICE) in the United Kingdom (https://www.nice.org.uk/), recommend the use of COS in their manual. However, to date, very little is known about the consideration and use of COS by guideline authors. We conducted a piece of research to examine whether or not COS were being referenced in guidelines and the extent to which the guideline outcomes selected agreed with outcomes listed in a relevant COS.^3^ We focussed on 10 disease areas with a published high‐quality COS and 38 guidelines with matching scope. What we found is that none of those guidelines explicitly mentioned or referenced the COS, and the agreement between the guideline outcomes and COS outcomes was variable. Although limited in terms of disease groups and geographical coverage, this exercise tells us that guideline authors are not yet routinely considering COS when choosing outcomes for their PICO statements (PICO is a framework to construct clinical questions and stands for Population, Intervention, Comparator and Outcome).

These findings were presented at the 2023 GIN conference in Glasgow and the response from the guideline community was very encouraging. We ran a workshop introducing the COMET database and discussing how to match the scope of a COS to a guideline question. It was standing room only. It seems that there is an appetite among guideline developers to find out more about COS and their relevance.

We know that when the scope for the NICE hip fracture guideline^4^ went out for consultation, a respondent (a member of the hip fracture COS team) criticised the fact the guideline development team had not considered the COS.^5^ The COS was then written into the guideline PICO. This case suggests that in some cases the barrier to guideline developers in using a COS could relate to a lack of awareness.

However, anecdotally, we hear that some guideline developers are dismissing COS that do not fully match their guideline in terms of population or condition. When a COS matches their scope at least partially, we would urge them to consider the list of outcomes in the COS and their potential relevance. Using a COS as a starting point for discussion with stakeholders, to then be adopted or adapted, is likely to be more efficient than starting from scratch. Some may feel that the list of outcomes in the COS is too limited as they have their own specialist insights about what is important; bear in mind that the COS is only ever intended as the *minimum* set of outcomes to be reported and authors are likely to supplement that list according to the context. Note, for example, that outcomes relating to resource utility would be an expected consideration for practice guidelines but are unlikely to appear in a COS for trials.

There is an increasing number of COS developed specifically for routine care,^6^ and recently many COS authors are working towards a COS that considers both research and routine care simultaneously. Addressing guideline use earlier in the COS pipeline, for example, by including guideline developers and healthcare commissioners in the consensus process, could be one way to ensure that the COS is fit for multiple purposes thus reducing research waste. During the COS‐STAD consensus process,^1^ the stakeholder group ‘Those who will use the research that should have used the COS (e.g., systematic reviewers, guideline developers, policy makers, regulatory agencies)’ narrowly missed the pre‐specified criterion for including it as minimum standard. Systematic reviews of published COS^2^ have reported an increase in the involvement of the end‐users of research; out of 54 COS development studies published in 2021, 19 (35%) had involved authority representatives (e.g., regulatory agencies, government agencies, policy makers) in their consensus process.

Uptake of COS in guidelines is likely to take time, just as it has done in trials, systematic reviews and other activities such as health technology assessments and regulatory guidance.^7^ We are keen to explore routes to increase consideration of COS by guideline developers. Please do not hesitate to get in touch with the corresponding author if you would like to discuss how best to do this or get involved in future initiatives related to COS and guideline development.

## CONFLICT OF INTEREST STATEMENT

P. W. chairs the COMET Management Group. I. D. F. leads the AGREE collaboration and he is Editor of ‘Clinical and Public Health Guidelines’.

## Data Availability

Data sharing is not applicable to this article as no new data were created or analysed in this study.
